# A review of available software for adaptive clinical trial design

**DOI:** 10.1177/1740774520906398

**Published:** 2020-02-17

**Authors:** Michael John Grayling, Graham Mark Wheeler

**Affiliations:** 1 MRC Biostatistics Unit, University of Cambridge, Cambridge, UK; 2 Population Health Sciences Institute, Newcastle University, Newcastle upon Tyne, UK; 3 Cancer Research UK & UCL Cancer Trials Centre, University College London, London, UK

**Keywords:** Code, dose escalation, group sequential, multi-stage, sample size re-estimation, phase I/II, phase II/III

## Abstract

**Background/aims:**

The increasing cost of the drug development process has seen interest in the use of adaptive trial designs grow substantially. Accordingly, much research has been conducted to identify barriers to increasing the use of adaptive designs in practice. Several articles have argued that the availability of user-friendly software will be an important step in making adaptive designs easier to implement. Therefore, we present a review of the current state of software availability for adaptive trial design.

**Methods:**

We review articles from 31 journals published in 2013–2017 that relate to methodology for adaptive trials to assess how often code and software for implementing novel adaptive designs is made available at the time of publication. We contrast our findings against these journals’ policies on code distribution. We also search popular code repositories, such as Comprehensive R Archive Network and GitHub, to identify further existing user-contributed software for adaptive designs. From this, we are able to direct interested parties toward solutions for their problem of interest.

**Results:**

Only 30% of included articles made their code available in some form. In many instances, articles published in journals that had mandatory requirements on code provision still did not make code available. There are several areas in which available software is currently limited or saturated. In particular, many packages are available to address group sequential design, but comparatively little code is present in the public domain to determine biomarker-guided adaptive designs.

**Conclusions:**

There is much room for improvement in the provision of software alongside adaptive design publications. In addition, while progress has been made, well-established software for various types of trial adaptation remains sparsely available.

## Introduction

Classically, clinical trials have used fixed-sample designs. In this approach, a trial is designed, carried out using the design, and the acquired data are analyzed on trial conclusion. In recent years, however, stagnation in the number of products submitted for regulatory approval^1^ and escalating drug development costs^2^ have led the trials community to seek new solutions to improving the efficiency of clinical research. One suggestion that has received much attention is that adaptive designs (ADs), which permit data-dependent modifications to be made to a trial’s conduct through a series of prospectively planned interim analyses, should be used more often. Indeed, both the U.S. Food and Drug Administration and the European Medicines Agency have recognized that ADs could become key in drug development.^3,4^

Subsequently, there has been an expansion in the publication of statistical methodology for the AD of clinical trials. Overviews can be found in several recent monographs.^5–7^ Furthermore, guidance is now available on when and why ADs may be useful, as well as on how to run such studies.^8–11^ Recommendations on how to report adaptively designed clinical trials are also under development.^12^

However, the actual number of trials that have used ADs remains small: a review of phase II, phase II/III, and phase III trials registered on ClinicalTrals.gov between 29 February 2000 and 1 June 2014, along with trials from the National Institute for Health Research register, identified only 143 AD clinical trials.^13^ A similar review of articles from several databases published prior to September 2014 found just 142 AD phase II, phase II/III, or phase III trials.^14^

Accordingly, much research has been conducted to identify and describe the potential barriers to the use of ADs.^15–24^ Many have since been identified. For example, a lack of available expertise in AD, the requisite length of time required for trial design when using an AD, a fear of AD would introduce operational biases, and inadequate funding structures. Our focus is on an additional barrier, which has been noted by several authors: a lack of easily accessible, well-documented, user-friendly software for AD.^15–20,23^ The provision of software for ADs is particularly important because, relative to fixed-sample designs, the complexity of ADs makes computational investigation of such methods typically a necessity. With the proliferation of software, it has been argued, project teams will be empowered to make informed decisions about the best design for their trial, and ultimately the frequency of appropriate AD use will increase. There have been recommendations that, wherever possible, software for novel AD methodology should be made available alongside statistical publications.^18^

Fortunately, therefore, several reviews of available software for ADs have been presented, providing either a focus on group sequential design,^25^ or a general overview.^6,26,27^ However, each of these concentrated on describing *what* software is available, focusing on established packages from a high-level perspective, giving particular attention to stand-alone proprietary solutions.

Here, our focus is directed toward two different aims. The first is to investigate the provision of user-contributed code and software for ADs in scientific publications. We review articles from a variety of journals that publish AD methodology, assessing how often code/software are provided alongside publications and how these results compare with the policies of these journals. Second, by searching several databases (including Comprehensive R Archive Network (CRAN), Statistical Software Components archive, and GitHub), we assess which AD features are supported by currently available user-contributed programs, focusing our attention on several programming languages that are popular in the trials community.

## Methods

### Review protocol

Here, we summarize the key points behind our literature and repository reviews. Further details are given in Supplementary File 1.

### Review aims

1. To determine the frequency with which requisite computer code is made available alongside publications relating to the AD of clinical trials, classifying this availability according to the archiving method and code completeness.
2. To determine the most popular programming languages used within the AD community.
3. To determine the degree to which authors who state computer code is “available upon request” are able to respond with said code following an e-mail request.
4. To identify and describe user-written code relating to the AD of clinical trials.

### Identification of relevant journal publications

#### PubMed Central search

PubMed Central was searched on 5 July 2018 by M.J.G. to identify potential publications for inclusion in our review. Articles were required to have been published in 1 of 31 journals; a bespoke selection we believed to be most likely to publish articles relating to AD methodology. Publications from each journal were identified by searching the [Abstract], [Body—Key Terms], and [Title] fields for 53 AD-related terms. The search was limited to articles published between 1 January 2013 and 31 December 2017. In total 4123 articles were identified for review.

#### Inclusion criteria

We desired to include publications related to the design and analysis of AD clinical trials. Thus, our inclusion criteria were:

1. A publication that proposes or examines design or analysis methodology for a clinical that “allows for prospectively planned modifications to one or more aspects of the design based on accumulating data from subjects in the trial”;^4^
2. A complete peer-reviewed publication (i.e. we excluded conference abstracts);
3. Set within the context of clinical trials (i.e. we excluded methodology that could be used for the AD of a clinical trial if the primary motivation was not clinical trial research);
4. Performs computational work of any kind relating to ADs (even to confirm theoretical results, produce simple graphs, etc.).

We excluded conference abstracts as we believed it would be unlikely that they would note whether/where code is available. Similarly, in fields other than clinical trials there may be different expectations on code availability. We thus excluded such publications to reduce the bias in our findings. No restrictions were made on the level of code required for inclusion since we felt drawing such conclusions would be subjective. Finally, note that by criterion 1, we excluded publications that simply presented the results of a trial that utilized an AD.

#### Selection of studies and data extraction

Two hundred records were randomly selected to pilot the selection process and data extraction upon. M.J.G. and G.M.W. independently considered the 200 records and for each of those marked for inclusion extracted the following data: Software availability (each of the articles were allocated into one of the categories given in Supplementary Table 2, according to their provision of code); software languages (R, SAS, Stata, Unclear, etc.)

Following this pilot, areas of disagreement were discussed in order to enhance the reliability of the selection process and data extraction on the remaining 3923 records, which were allocated evenly and at random to M.J.G. and G.M.W. In extreme cases where a reviewer was unable to come to a conclusion on inclusion/data extraction, a decision was made following discussion with the other reviewer.

### Identification of relevant database-archived computer code

#### Software-specific database searches

To identify further available software for AD, M.J.G. performed the following additional software-specific database searches on 10 July 2018. For each, there was no simple means of extracting results data into a manageable offline form. Therefore, a less formal approach to record identification had to be taken.

First, Rseek was used to identify pertinent packages available on the CRAN (the principal location for the storage of R packages). Each of the 53 terms used in the article search of PubMed Central (the “search terms”) were entered into the search engine at https://rseek.org/. Next, the articles from the R-project tab were screened, with any that appeared to be of potential relevance to ADs noted in a .csv file. Similarly, to identify code available for Stata that is relevant to ADs, the Statistical Software Components archive was used (which hosts the largest collection of user-contributed Stata programs). The search terms were entered into the search bar at https://ideas.repec.org/. Any potentially germane results were added to the aforementioned .csv file. The search terms were also entered into the search engine at https://www.stata-journal.com/, in order to identify relevant publications in the *Stata Journal* (note: we did not search for *Stata Journal* articles via PubMed Central, as not all such articles are indexed there), the premier journal for the publication of Stata code articles. To find user-contributed code for AD in SAS, the abstracts of the proceedings of the SAS Global Forums from 2007 to 2018 were searched using the search terms (e.g. for 2016 the terms were utilized via Ctrl+F searches at support.sas.com/resources/papers/proceedings16/). Finally, the procedure was repeated on GitHub, using the search bar at https://github.com/. For this search, no restrictions on the programming language utilized were made.

Note that for each of these databases, no limits on the publication date were employed. The number of records identified of potential relevance are given in Supplementary Table 3.

#### Identification of relevant records

Each of the records were assessed to identify those related to ADs. Our criterion for listing a record as relevant was criterion 1 from the “Inclusion criteria” section. The functionalities of those that were relevant were noted via a checklist, using the following keywords: AD type (Adaptive randomization; Bayesian methods; Biomarker-based methods; Dose-modification/escalation; Group sequential; Sample size adjustment); AD features (Alpha spending; Drop the loser; Multi-stage; Pick the winner; Stopping rules; Two stage); Phase type (Phase I; Phase I/II; Phase II; Phase II/III; Phase III).

In addition, we extracted data on several descriptors that could be viewed as indicators of the quality or potential ease-of-use of relevant records in practice. These are whether records relate to a software package or simply a collection of functions; include help files; include a vignette/guide (or other long-form documentation) or are associated with a published article (e.g. in *Journal of Statistical Software*); depend on other unvalidated software (e.g. an R package may depend on other R packages that are not part of base R); contain annotated code.

To pilot the screening, 31 records (∼10% of the 307 records initially identified) were chosen at random and reviewed by M.J.G. and G.M.W. As above, this allowed for discussions on differences of opinion, to improve the classification of the remaining records. For efficiency purposes, M.J.G. then screened each of the remaining records from GitHub. G.M.W. screened those from each of the other databases.

Note that for all records that were marked to be of relevance, the author’s additional repositories were screened (e.g. via their homepage on GitHub). From this, three previously unidentified records were included.

## Results

### Code availability

Our search yielded 4123 articles across the 31 considered journals (Supplementary Table 1). Of these, 3875 were excluded on the following grounds: Non-adaptive design methodology (3817); Not a complete peer-reviewed article (40); Not directly applied to clinical trials (13); No computational work required (6). This left 247 eligible articles from 26 journals for further review.

Of these 247 articles, 144 (58.3%) did not provide code. Thirty-two articles (13%) provided complete code in the article or its supplementary material to either recreate the exact outputs of the article, or provided all functions to do so; a further 8 articles (3.2%) provided partial code. Twenty-seven articles (10.9%) provided URL addresses to websites where code was to be stored; of these only 13 (48%) were accessible at the time of review. The remainder either did not provide the code for the relevant article or the URL no longer worked. Six articles (2.4%) stated that code was available in online supplementary material, but the code was not present. In another six articles (2.4%), code was either released as a downloadable package/standalone software (4/6), or the functions were incorporated into an existing package (2/6). One article cited software that could be used for the purpose it outlined, but no further details were provided, and another used commercial software for their work so no code/instructions were provided.

The remaining 22 articles (8.9%) stated that code was available upon request from the corresponding author. For all 22 articles we sent request e-mails to the corresponding author, explaining that their article stated code was available upon request and that we were asking for it as part of a literature review on AD code availability (e-mail template given in Supplementary File 1). Authors were given 1 month from the date of e-mail to reply and were sent a reminder e-mail 2 weeks after first contact. From these 22 requests, 14 (63.6%) authors replied to either provide the code used, or to direct us to a URL where the code was deposited; one author replied to say that the code was not available. Six authors (27.3%) did not reply to our request. One author was uncontactable via their stated e-mail address; a search for an up-to-date address yielded no leads.

**Figure 1.**
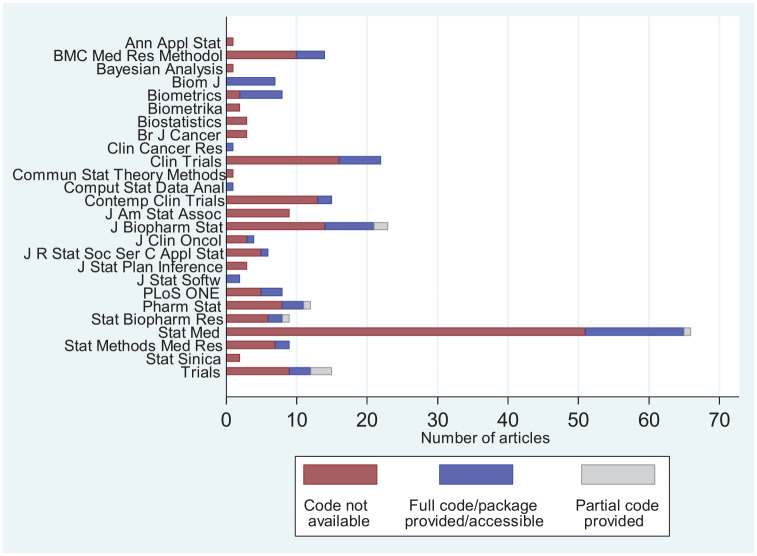
Number of articles by journal and whether code is provided or not. Note that only articles published between 1 January 2013 and 31 December 2017 were reviewed.

Incorporating the author responses that provided code or accessible URLs to code into our results, code was accessible (either directly in the article, via a valid URL, or incorporated into an available software package) for 65 articles (26.3%), with a further eight articles (3.2%) providing partial code. The remaining 174 articles (70.4%) did not provide code relating to the proposed AD methodology. Figure 1 shows the distribution of code provision by journal.

### Code availability and journal policies

**Figure 2.**
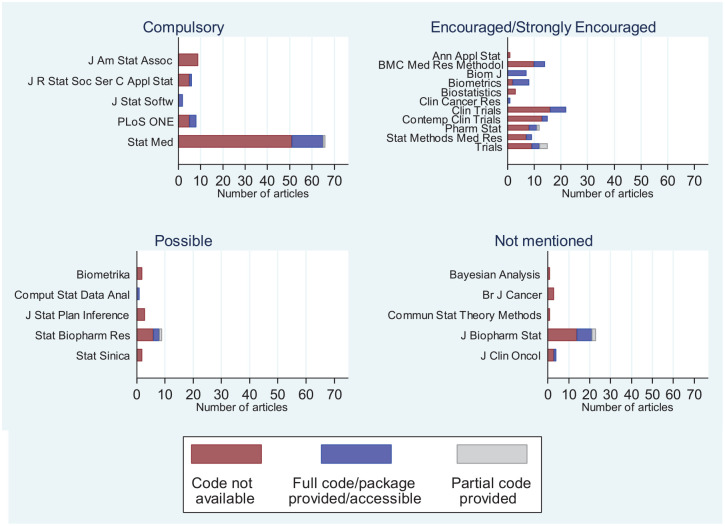
Number of articles by code provision, journal and journal’s code provision policy. Note that only articles published between 1 January 2013 and 31 December 2017 were reviewed, and data on journal policies were extracted on 22 October 2018.

Policies on whether computer code should be provided with article submissions vary between journals. Therefore, we reviewed journal policies on providing computer code and compared them to the observed rates of code availability/provision in our review. At https://github.com/mjg211/article_code we provide a Microsoft Excel file that details the journal policies on code provision along with their categorization (Compulsory, Strongly Encouraged, Encouraged, Possible, Not Mentioned; data on journal policies extracted on 22 October 2018) and Figure 2 shows the distribution of code provision across journals according to their code provision policy. The data show journals with compulsory policies for code provision have not been enforcing their policies.

There is a possibility that articles published at the start of our review period (i.e. 2013) may not have been subject to the same code provision policy that is in place now. However, violations of the compulsory policy type are consistent across the review period. For example, *Statistics in Medicine* (ISSN 0277-6715; Wiley Online Library) states that “The journal also *requires* authors to supply any supporting computer code or simulations that allow readers to institute any new methodology proposed in the published article”; this is an example of a compulsory policy. In our review, 66 articles published in *Statistics in Medicine* were considered eligible. Table 1 shows the distribution of articles published each year across journal provision type for articles published in *Statistics in Medicine*. Over 5 years, 51 articles (77%) were published with no code provided, and numbers did not noticeably decrease over time, which would be consistent with the introduction of a compulsory code provision policy.

**Table 1.** Code provision for articles published in Statistics in Medicine, split by year of first publication.

	Year of publication					Total
	2013	2014	2015	2016	2017	
Code notavailable	4	12	13	12	10	51
Full code/packageprovided/accessible	1	3	2	3	5	14
Partial codeprovided	0	0	0	0	1	1
Total	5	15	15	15	16	66

### Software used

A variety of different statistical programs were used in the eligible articles, including open-source libraries, licensed programs, and commercial software. Overall, 129 articles (52%) stated what software was used in their computations; 60 of these articles (47%) did not make their code available.

Of the 129 articles, 107 used R;^28^ 91 such articles used R only, and the other 16 used R in combination with another program (e.g. MCMC sampling software such as JAGS, OpenBUGS, or WinBUGS), or provided code/software in other computing languages as well as R. Table 2 shows the usage of different software and their provision categories in journals.

**Table 2.** Software used in adaptive design articles (where stated) across code provision category.

	Code notavailable	Full code/packageprovided/accessible	Partial codeprovided	Total
**C**	1	2	–	3
**Excel**	–	2	–	2
**FACTS**	2	1	–	3
**FORTRAN**	1	2	–	3
**JAGS**	1	–	1	2
**Matlab**	–	2	–	2
**PASS**	1	–	–	1
**R** ^a^	52	49	6	107
**EAST**	–	–	1	1
**SCPRT**	–	–	1	1
**OpenBUGS**	1	–	–	1
**WinBUGS**	1	1	3	5
**Stand-aloneprogram**	1	7	–	8
**P3M**	1	–	–	1
**Stata**	2	2	1	5
**SAS**	2	3	–	5

^a^Includes custom R functions, use of existing R packages, and also R Shiny applications.

### Repository review

We performed additional searches of major software libraries to identify and classify available software related to ADs. Our searches identified 310 records, of which 122 were considered eligible. Of these, 64 (52%) were found on CRAN; 45 of these 64 CRAN packages had duplicate repositories on GitHub pages. Forty (33%) additional repositories were found on GitHub (i.e. repositories not located on any other platform), 8 (7%) on Statistical Software Components archive, 6 (5%) from the SAS Global Forum and 4 (3%) from the *Stata Journal*. Of the 40 GitHub repositories, 35 (88%) featured code for R; the remaining 5 entries featured code for Julia (2/40), JavaScript (1/40), Python (1/40), and SAS (1/40). This means that of the 122 eligible repositories, 99 (81%) provided R packages or code for use in R.

Table 3 shows the primary applications for AD software, split by software language and intended trial phase. The applications for AD software fell into at least one of the six “AD type” categories stated in the “Identification of relevant records” section. The majority of available software covers phase II and phase III trials and are for group sequential methods. The packages/programs tended to cover multiple purposes; 63 programs belonged to one of the design categories listed in Table 3, 54 belonged to two categories, and 5 belonged to three categories.

**Table 3.** Main functions of software repositories, split by software and trial phase.

	Groupsequentialmethods	Dosemodification/escalation	Sample size adjustment	Adaptive randomization	Bayesianmethods	Biomarker-basedmethods
**Software**						
** JavaScript**	–	1	–	–	–	–
** Julia**	1	–	1	1	–	1
** Python**	1	–	–	–	1	–
** R**	48	36	9	8	45	9
** SAS**	3	1	–	4	1	1
** Stata**	9	1	1	–	3	–
**Phase**						
** I**	–	27	–	–	21	–
** I/II**	–	10	–	–	8	1
** II**	56	10	10	13	27	10
** II/III**	2	1	–	–	1	–
** III**	46	2	9	12	14	7

Each package may belong to multiple categories and cover multiple trial phases.

Supplementary Table 4 shows the distribution of software and trial phase catered for by the different AD features (as described in the “Identification of relevant records” section). When assessing these AD features, the vast majority are included as part of packages for group sequential designs. Most of these packages cater for two-stage and multi-stage designs, and allow for early-stopping rules. As per previous tables, R is generally the favored language for writing such programs.

**Figure 3.**
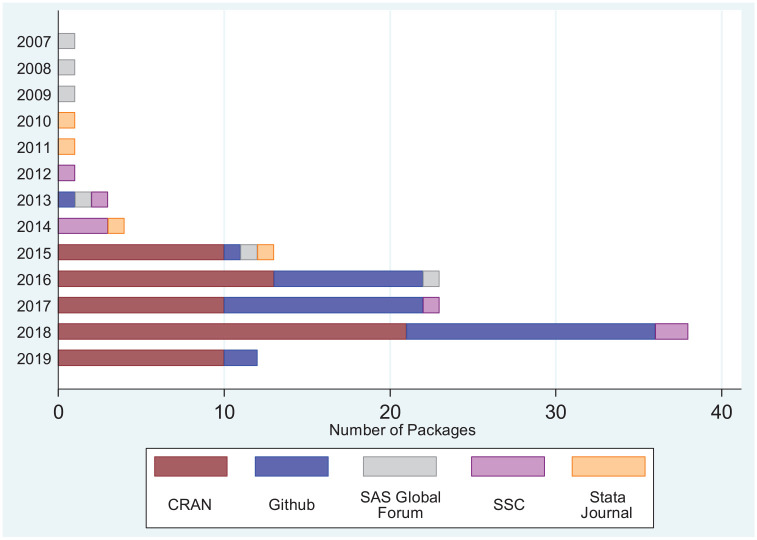
Number of identified repositories by location and year of last modification. Note that repositories were identified through searches conducted on 10 July 2018, while data on date of last modification were extracted on 15 March 2019.

We also extracted on 15 March 2019, where possible, the date when the software was last modified or released. For 10 (8%) entries, only the year of last known update was available. Figure 3 shows the distribution of year of latest modification by storage location (e.g. CRAN). Most packages are hosted on CRAN and GitHub, repositories that users can easily update and submit packages to, and all CRAN packages have been released or updated within the last 4 years. There are few programs hosted on the SAS Global Forum, Statistical Software Components archive, and via the *Stata Journal*, most of which have not been updated in the last 4 years. We cannot tell if the lack of updates is because the package is in perfect working order with all required functionality, or whether a lack of interest means there is no need for the maintainer to provide updates.

Finally, Supplementary Table 5 summarizes the extracted data on the software quality descriptors. We see that 94 of the 122 included records related to a software package (77%), which may in general ease installation and thus use. In addition, 95 records made help files available (78%). However, only 42 had associated long-form documentation (34%), 88 (72%) depended on other unvalidated software, and just 16 (13%) were viewed to have well annotated their code.

## Discussion

By scanning 31 journals and 5 years’ worth of publications, we provide reliable estimates of the prevalence of software provision alongside AD methodology publications. The reliability of our findings is also aided by joint-review of an initial subset of records, with discussion of findings to ensure consistency. Ultimately, we found that 70% of included articles did not provide any code or software. Most of the journals in which these articles were published have code provision policies that either require or strongly encourage the provision of code.

The low rate of software provision is a disappointing finding. Providing code alongside methodological research allows readers to reproduce novel ADs and tailor them to their own project needs. Some research funders expect funding recipients to make data and original software used for analyses fully available at the time of publication. For example, the Wellcome Trust state that researchers should make sure such outputs (a) are discoverable, (b) use recognized community repositories, and (c) use persistent identifiers (e.g. DOIs) where possible (see https://wellcome.ac.uk/funding/guidance/policy-data-software-materials-management-and-sharing). We recommend that this guidance is followed for all AD-related publications whenever feasible.

A related important point is that our findings are likely to be indicative of a wider problem in biostatistics. We are unaware of any article that has examined code availability in other fields, and an argument could be made that the typical increased complexity of code for AD may be inhibiting its distribution. However, it seems reasonable to assume that code provision at the time of publication may be poor across many areas of biostatistical research. Consequently, further guidance from journals on code requirements, or from group-based consensus recommendations on best practice, appear warranted.

More positively, we identified that there has been a marked increase in the number of software repositories relating to ADs over the last 5 years (Figure 3). A further interesting result is that the majority of AD-related programs are written for R. Therefore, while provision of code and software with new publications may help increase the use of ADs, it would also thus be prudent for statisticians to be familiar with how to use R. Furthermore, by demonstrating what trial adaptations are covered by existing software, we have made it possible for researchers to be better informed as to where new and improved code is required. In particular, many programs are available for group sequential design. In contrast, only limited software is available to support sample size re-estimation, or biomarker-based adaptation.

Although it is not the focus of our work, it feels appropriate to comment on more general issues relating to user-contributed code. The first issue relates to how researchers can identify available code for their problem of interest. For this, additional detailed articles that provide updates to previous works^6,25–27^ would likely have notable value to the trials community. So too would an online curated database on AD-related software (similar to the CRAN Task Views maintained for R). At present, there is no simple means for a researcher to access information on the currently available tools for AD. However, we do note that in Supplementary File 1 we both provide overviews, based on our experiences, of the packages we believe to have the greatest utility for each type of adaptation. In addition, we provide an example of how our dataset could be used to identify potentially useful code.

The second issue relates to the key problem of the quality, and therefore likely the applicability in practice, of user-contributed code. In almost all cases, such code will be unvalidated and come with no guarantees. Therefore, it is important that researchers realize substantial time and effort may be required to validate such software before it could be used to design a particular trial. Accordingly, the provision of help files, package documentation, and comprehensive code annotation should be greatly appreciated. Furthermore, the development of new tools to help with software validation will also be of much value. To this end, projects such as rOpenSci, along with newly established journals such as *Journal of Open Source Software* and *Software Impacts*, provide interesting potential routes to having code critiqued to improve its quality. We encourage researchers to embrace such tools to help improve the quality of available user-contributed code over time.

Finally, we note limitations of our work. First, some articles may not release code at the time of publication as they intend to release their code as part of a larger package, or because of potential confidentiality issues. However, no article mentioned that this was the case, and we would encourage authors to state why code is not available. In addition, we captured only a snapshot in time of repositories that were identifiable using our chosen review process. Consequently, it is likely numerous other repositories are available relating to AD, and the dataset we have made available should not be viewed as a list of all code that can currently be accessed.

In summary, to overcome the barriers to implementing ADs in clinical trials, we encourage researchers to make their code available alongside their published research, or by storing it on stable repositories. Several articles stated code was available at a given URL, but half of these URLs did not work. Similarly, around one third of articles that stated code would be available upon request were unable to provide code within a month of sending a written request. Therefore, making code available in either of these manners should not be viewed as a reliable long-term method of user access.

## Supplemental Material

Supplemental_Material – Supplemental material for A review of available software for adaptive clinical trial design Click here for additional data file. Supplemental material, Supplemental_Material for A review of available software for adaptive clinical trial design by Michael John Grayling and Graham Mark Wheeler in Clinical Trials
