# Supplementary material for: A review of available software for adaptive clinical trial design
Source: Clin Trials. 2020 Feb 17;17(3):323–31. doi: 10.1177/1740774520906398 (PMC7736777; doi:10.1177/1740774520906398)
Supplement: Supplemental_Material – Supplemental material for A review of available software for adaptive clinical trial design [file Supplemental_Material.pdf]

Supplementary File 1 to 'A review of available software for adaptive clinical trial design'

PubMed Central search

The following 53 search terms were used in relation to the PubMed Central search described in Section 2.3.1 of the main manuscript

Adaptive approach; adaptive clinical; adaptive design; adaptive method; adaptive randomisation; adaptive randomization; adaptive rule; adaptive trial; alpha spending; bayesian adaptive; bayesian approach; bayesian clinical; bayesian design; bayesian method; bayesian model; bayesian rule; bayesian trial; dose-escalation; dose-selection; dose escalation; dose finding; dose selection; drop the loser; flexible design; group sequential; interim analysis; multi-stage; multi stage; phase 1; phase 1-2; phase 1/2; phase 2; phase 2-3; phase 2/3; phase 3; phase I; phase I-II; phase I/II; phase II; phase II/III; phase III; pick the winner; play the winner; response adaptive; sample adjust; sample re-estimation; sample reestimation; sample size adjust; sample size re-estimation; sample size reestimation; stopping rule; two-stage; two stage.

Note that the following term-field combinations were found to be absent from PubMed Central, and so were subsequently omitted from the search text

adaptive clinical[Abstract]; bayesian clinical[Abstract]; bayesian trial[Abstract]; sample adjust[Abstract]; sample re-estimation[Abstract]; sample reestimation[Abstract]; sample size adjust[Abstract]; bayesian clinical[Body - Key Terms]; drop the loser[Body - Key Terms]; phase 2/3[Body - Key Terms]; phase II-III[Body - Key Terms]; pick the winner[Body - Key Terms]; sample adjust[Body - Key Terms]; sample re-estimation[Body - Key Terms]; adaptive clinical[Title]; adaptive randomisation[Title]; bayesian trial[Title]; bayesian clinical[Title]; sample re-estimation[Title]; sample size adjust[Title]; sample adjust[Title]; play the winner[Title]; sample reestimation[Title].

Thus, as an example, the extracted records for the *Journal of Statistical Software* were found with the following search

(((
(((((((multi-stage[Abstract]) OR multi-stage[Body - Key Terms]) OR multi-stage[Title]) OR multi stage[Abstract]) OR multi stage[Body - Key Terms]) OR multi stage[Title]) OR adaptive design[Abstract]) OR adaptive trial[Abstract]) OR adaptive rule[Abstract]) OR adaptive method[Abstract]) OR adaptive approach[Abstract]) OR adaptive randomisation[Abstract]) OR adaptive randomization[Abstract]) OR flexible design[Abstract]) OR group sequential[Abstract]) OR bayesian design[Abstract]) OR bayesian adaptive[Abstract]) OR bayesian model[Abstract]) OR bayesian approach[Abstract]) OR bayesian rule[Abstract]) OR bayesian method[Abstract]) OR two stage[Abstract]) OR interim analysis[Abstract]) OR sample size re-estimation[Abstract]) OR stopping rule[Abstract]) OR drop the loser[Abstract]) OR pick the winner[Abstract]) OR play the winner[Abstract]) OR dose selection[Abstract]) OR dose-selection[Abstract]) OR dose escalation[Abstract]) OR dose-escalation[Abstract]) OR dose finding[Abstract]) OR phase 2-3[Abstract]) OR phase 2/3[Abstract]) OR phase II-III[Abstract]) OR phase II/III[Abstract]) OR phase 1-2[Abstract]) OR phase 1/2[Abstract]) OR phase I-II[Abstract]) OR phase I/II[Abstract]) OR alpha spending[Abstract]) OR response adaptive[Abstract]) OR phase I[Abstract]) OR phase II[Abstract]) OR phase III[Abstract]) OR phase 1[Abstract]) OR phase 2[Abstract]) OR phase 3[Abstract]) OR adaptive design[Body - Key Terms]) OR adaptive trial[Body - Key Terms]) OR adaptive clinical[Body - Key Terms]) OR adaptive rule[Body - Key Terms]) OR adaptive method[Body - Key Terms]) OR adaptive approach[Body - Key Terms]) OR adaptive randomisation[Body - Key Terms]) OR adaptive randomization[Body - Key Terms]) OR flexible design[Body - Key Terms]) OR group sequential[Body -

Key Terms]) OR bayesian design[Body - Key Terms]) OR bayesian adaptive[Body - Key Terms]) OR bayesian model[Body - Key Terms]) OR bayesian approach[Body - Key Terms]) OR bayesian trial[Body - Key Terms]) OR bayesian rule[Body - Key Terms]) OR bayesian method[Body - Key Terms]) OR two stage[Body - Key Terms]) OR interim analysis[Body - Key Terms]) OR sample size re-estimation[Body - Key Terms]) OR sample size adjust[Body - Key Terms]) OR stopping rule[Body - Key Terms]) OR play the winner[Body - Key Terms]) OR dose selection[Body - Key Terms]) OR dose-selection[Body - Key Terms]) OR dose escalation[Body - Key Terms]) OR dose-escalation[Body - Key Terms]) OR dose finding[Body - Key Terms]) OR phase 2-3[Body - Key Terms]) OR phase II/III[Body - Key Terms]) OR phase 1-2[Body - Key Terms]) OR phase 1/2[Body - Key Terms]) OR phase I-II[Body - Key Terms]) OR phase I/II[Body - Key Terms]) OR alpha spending[Body - Key Terms]) OR response adaptive[Body - Key Terms]) OR phase 1[Body - Key Terms]) OR phase 2[Body - Key Terms]) OR phase 3[Body - Key Terms]) OR phase I[Body - Key Terms]) OR phase II[Body - Key Terms]) OR phase III[Body - Key Terms]) OR adaptive design[Title]) OR adaptive trial[Title]) OR adaptive rule[Title]) OR adaptive method[Title]) OR adaptive approach[Title]) OR adaptive randomization[Title]) OR flexible design[Title]) OR group sequential[Title]) OR bayesian design[Title]) OR bayesian adaptive[Title]) OR bayesian model[Title]) OR bayesian approach[Title]) OR bayesian rule[Title]) OR bayesian method[Title]) OR two stage[Title]) OR interim analysis[Title]) OR sample size re-estimation[Title]) OR stopping rule[Title]) OR drop the loser[Title]) OR pick the winner[Title]) OR dose selection[Title]) OR dose-selection[Title]) OR dose escalation[Title]) OR dose-escalation[Title]) OR dose finding[Title]) OR phase 2-3[Title]) OR phase 2/3[Title]) OR phase II-III[Title]) OR phase II/III[Title]) OR phase 1-2[Title]) OR phase 1/2[Title]) OR phase I-II[Title]) OR phase I/II[Title]) OR alpha spending[Title]) OR response adaptive[Title]) OR phase 1[Title]) OR phase 2[Title]) OR phase 3[Title]) OR phase I[Title]) OR phase II[Title]) OR phase III[Title]) OR two-stage[Abstract]) OR two-stage[Body - Key Terms]) OR two-stage[Title]) OR sample size reestimation[Abstract]) OR sample size reestimation[Body - Key Terms]) OR sample size reestimation[Title]) OR sample reestimation[Body - Key Terms]) AND ("2013/01/01"[Publication Date] : "2017/12/31"[Publication Date])) AND "Journal of Statistical Software"[Journal]))

For those articles which were included, Supplementary Table 2 then provides further information on the possible classification of the level of code provision.

In addition, note that in each case of record exclusion, a reason for exclusion amongst the following options was recorded:

- Non-adaptive design methodology;
- No code required;
- Not within the context of clinical trials;
- Not complete publication.

E-mail template for code requests

Subject: Request for computer code: <authors> (<journal>, <year>)

Body:

Dear <author>,

We are two UK researchers conducting a survey on the provision of software and computer code in academic publications, specifically focusing on adaptive designs in clinical trials. One aim of this work is to assess the availability of software and code for researchers in adaptive designs, and how complete any available code is.

One of the eligible studies in our survey was the paper “<article title>” (<authors>, <journal>, <volume>(<issue>):<pages>. <year>), for which you are the corresponding author. In this article, it says <text stating code is available upon request>. For eligible articles that say code is available upon request, we are e-mailing the corresponding author to request the code used.

Would you be able to provide us with the code used to generate the results and figures in this paper? If so, please send all relevant and available code files to Dr. Graham Wheeler (graham.wheeler@ucl.ac.uk) by <date one month from date e-mail is to be sent> at the latest.

If it is not possible to provide us with any of the code files used, please do reply informing us of this.

Yours sincerely,

Dr. Graham Wheeler, CRUK and UCL Cancer Trials Centre, University College London, UK (graham.wheeler@ucl.ac.uk)
Dr. Michael Grayling, Institute of Health and Society, University of Newcastle, UK (michael.grayling@newcastle.ac.uk)

Database searches

Supplementary Table 3 provides information on the number of records identified in each of the considered databases, whilst Supplementary Table 4 shows the distribution of software and trial phase catered for by the different subcategories of group sequential methods.

Description of Microsoft Excel file

Further supplementary material, in the form of an .xlsx file, is made available at https://github.com/mjg211/article_code. This file contains the following three Microsoft Excel sheets:

- *Article review:* In this sheet, details on the 4123 that were extracted from PubMed Central and reviewed as part of our discussed assessment of code provision alongside AD-related publications are given. In particular, the final eleven columns indicate who reviewed the article, whether it was included in the final analysis, the reason for exclusion, and the software language used, as was relevant.
- *Journal policies:* This sheet describes the extracted code provision policies of the 26 journals for which at least one article was included in our article review. In particular, in Column D, phrasing that led to the chosen categorization (Column C) is highlighted in red.
- *Repository review:* This sheet provides details on the 122 records that were included in our repository review. In particular, categorizations of each record for each of the keywords is given, along with data such as the last date of modification, and data on the various selected indicators of code quality. It is this sheet that is utilized below to identify potentially relevant code for an example trial design problem.

Using the repository review to identify relevant software

The Microsoft Excel file available at https://github.com/mjg211/article_code contains a sheet on the 122 code repositories that we identified which contained software relating to AD. In this section, we describe how researchers could use these details to identify potentially useful available code for a particular problem of interest to them.

Specifically, we consider wanting to design a phase I dose-escalation trial. Suppose that it is further stipulated that Bayesian methodology must be used and that there is a preference for programming in R. Within Microsoft Excel, we can identify potentially suitable records by sorting on the columns of the sheet 'Repository review' (see above). Precisely we apply the following custom sort:

- by column 'Dose modification/escalation', Z-A;
- then by column 'Phase I', Z-A;
- then by column 'Bayesian methods', Z-A;
- then by column 'Software', A-Z.

We find that 21 records are categorized as 'Yes' for each of the columns 'Dose modification/escalation', 'Phase I', and 'Bayesian methods', with 20 relating to software for R and one to software for Stata. Because numerous repositories are available that contain R code, we may discount the Stata related record to conform to the preference for using R.

Whilst each of these 20 records may of course suit the needs of the trial scenario under consideration, prioritizing which of the 20 records may be most likely to be useful could then be quickly achieved by examining the 'Source' column along with the code quality columns (Columns AG-AK). In this instance, 16 of the records are from CRAN and the remainder are from GitHub. We may in general anticipate code on CRAN to be more developed than that on GitHub, and so may narrow our focus to these 16 records.

We might next split the records to consider first those that do not have further dependencies on unvalidated software (of which there are only 3: CRM, dfcrm, and UBCRM). The 'Description' column could then be used as a simple indicator or whether any of these packages may be viewed as potentially useful for the trial scenario under consideration (according to any additional design restrictions that may be in place).

If they do not appear to meet the required needs, a sensible way to prioritize which packages to consider next could be the 'Vignette/package guide/associated article available' column, which reveals that there are six packages (with other dependencies) for which long-form documentation is available (modest, bcrm, BOIN, dfpk, crmPack, trialr). Again, these could then be quickly assessed using the 'Description' column before the remaining seven packages were considered for their utility.

Overview of available software by design adaptation

Introduction

Though our work focuses on (a) the frequency with which code is made available alongside AD related publications and (b) the current volume of code repositories relating to ADs, it also seems logical to provide short descriptions of packages that we have experience with, which we consequently feel may be amongst those with the broadest utility for researchers. In this section, we achieve this by providing overviews below by type of adaptation.

Group sequential methods

Much software is available to support group sequential trial design. Within R, arguably the most established package is gsDesign. A Bayesian version gsbDesign is also now available. Alternatively, the package GroupSeq provides similar functionality via a Graphical User Interface, whilst OptGS allows users to identify optimal group sequential designs. In Stata the module GROUPSEQ is available and has a supporting article describing its functionality in the *Stata Journal*.

Other relevant R packages include MAMS, for multi-arm multi-stage trial design, and OneArmPhaseTwoStudy, for group-sequential single-arm trial designs commonly utilized in phase II oncology. In Stata, similar functionality is provided by the commands nstage and simon2stage respectively.

Dose modification/escalation

Several packages are available for designing conducting dose-escalation studies, with the vast majority of these available as R packages from CRAN or GitHub repositories. The main R packages for single-agent dose-escalation studies include CRM, dfcrm, dfmta, bcrm, crmPack, modest and ewoc, many of which are updated on a regular basis. Other packages also cater for trials where two or more agents are combined (e.g., dfcomb, BOIN, pocrm).

For dose-finding in phase II, packages such as MCPMod and DoseFinding can be used. However, it is worth noting that the MCPMod package will not be further developed, and all future updates will be made via the DoseFinding package.

Sample size adjustment

We have first-hand knowledge of only a small amount of software relating to sample size adjustment, with none supporting for example commonly utilized internal pilot designs. Currently, gsDesign supports sample size re-estimation based on interim estimated conditional power, whilst a selection of packages enable adaptive two-stage single-arm designs to be identified (e.g., asimon for R and BinaryTwoStageDesigns for Julia). Perhaps most developed in this area is the R package Power2Stage, which can evaluate the operating characteristics of a wide array of two-stage Bioequivalence trial designs.

Adaptive randomization

We are aware of very little code that is currently available that supports response adaptive trial design. Thus, researchers considering using such a design should anticipate needing to utilize a proprietary software solution or needing to develop their own applicable code. The most notable exception to this is the R package BayesAdaptive. This package supports Bayesian response-adaptive designs for multi-arm trials, following the methodology presented in a referenced publication, includes detailed help files as well as a package vignette, and includes a large number of functions for designing such trials and evaluating their operating characteristics.

Bayesian methods

Many available software packages support the determination of Bayesian adaptive designs. Along with the numerous Bayesian phase I methods that are discussed above, as well as the packages BayesAdaptive, trialr (also supporting EffTox phase I/II design), and gsbDesign that have also already been mentioned, these notably include (all for R): ph2bye for identifying Bayesian sequential single-arm designs for phase II oncology trials, EffToxDesign for using the EffTox phase I/II design, and BACT for simulating and analyzing Bayesian adaptive trials.

Biomarker-based methods

As noted in main manuscript, appears very little is currently available for biomarker guided trial design. the most familiar relevant software of which the authors are familiar is ph2hetero, which supports

several methods for designing adaptively enriched single-arm phase II trials and has an associated article available describing its functionality in detail.

Supplementary Table 1. A list of the journals included in the search procedure, and the number of identified potential records for inclusion for each, is given.

Journal	Records
Annals of Statistics	4
Bayesian Analysis	10
Biometrical Journal	12
Biometrics	62
Biometrika	8
Biostatistics	21
BMC Medical Research Methodology	71
British Journal of Cancer	276
Clinical Cancer Research	282
Clinical Trials	55
Communications in Statistics: Theory and Methods	2
Computational Statistics & Data Analysis	6
Contemporary Clinical Trials	64
Journal of Biopharmaceutical Statistics	35
Journal of Clinical Oncology	288
Journal of Statistical Planning and Inference	6
Journal of Statistical Software	3
Journal of the American Statistical Association	41
Journal of the Royal Statistical Society. Series B, Statistical Methodology	4
Journal of the Royal Statistical Society. Series C, Applied Statistics	20
Pharmaceutical Statistics	14
PLoS ONE	2094
Statistica Sinica	7
Statistical Methods in Medical Research	41
Statistics & Probability Letters	1
Statistics in Biopharmaceutical Research	14
Statistics in Medicine	154
Technometrics	1
The Annals of Applied Statistics	25
The BMJ	26
Trials	476
Total	4123

Supplementary Table 2. Possible classifications on the provision of software of publications included in the review, and a more detailed description of their meaning, is provided.

Classification	Meaning
Included in paper/appendix – Complete code provided (to exactly reproduce results)	The relevant code is made available with the publication, either as part of the main paper or in an (potentially online) appendix. This code is an executable form that allows the results from the paper to be reproduced exactly
Included in paper/appendix – Complete code provided (functions only)	The relevant code is made available with the publication, either as part of the main paper or in an (potentially online) appendix. All functions that are in theory required to be able to reproduce the results from the paper are supplied, but not in an executable form for the exact production of results
Included in paper/appendix – Partially complete code provided	Relevant code is made available with the publication, either as part of the main paper or in an (potentially online) appendix. However, the code does not allow for all of the calculations in the publication to be replicated
Incorporated into previously released software/package	The relevant code is included as a new feature in a previously released software package
Released as stand-alone software/package	The relevant code is released as a stand-alone, referenced, and typically downloadable, software package
URL to website provided (still accessible)	A URL is given to where relevant code could be acquired, and the code remains accessible at this address
URL to website provided (no longer accessible)	A URL is given to where relevant code could be acquired. However, the code can no longer be accessed at this address
Made available upon request	Code is listed as being available upon e-mail request from one of the publication's authors
Not discussed/made available	The provision of relevant code is not discussed or made available in any of the forms given above
Other	Several articles did not fall in to any of the above classifications. A detailed description of why is provided for each in the online supplementary Microsoft Excel file, and in the main article

Supplementary Table 3. The number of identified potential records of relevance to the AD of clinical trials, from our search of software-specific database searches, is given.

Database	Records
GitHub	161
RSeek	103
SAS Global Forum	16
SSC	9
Stata Journal	21
Total	310

Supplementary Table 4. AD features broken down into constituent parts by software. Numbers also belonging to "Group sequential" AD type category shown in parentheses.

	Two-stage	Multi-stage	Stopping Rules	Drop the loser	Pick the winner	Alpha spending
JavaScript	-	-	-	-	-	-
Julia	1 (1)	-	1 (1)	-	-	-
Python	-	1 (1)	1 (1)	-	-	-
R	33 (27)	30 (26)	34 (26)	2 (2)	3 (3)	12 (12)
SAS	2 (2)	4 (1)	2 (1)	-	-	1 (1)
Stata	3 (3)	6 (6)	8 (7)	1 (1)	1 (1)	1 (1)

Supplementary Table 5. Record categorizations for the descriptors of code quality.

Descriptor	Categorisations		
	Full package	Functions not packaged together	Stand-alone application
Package, code, or application?	94	26	2
	Yes		No
Help files available?	95		27
Vignette/package guide/associated article available?	42		80
Depends on other unvalidated software?	88		34
	Well annotated	Partially annotated	Poorly annotated/unannotated
Contains annotated code?	16	25	81
